# Supplementary material for: Robust and prototypical immune responses toward COVID-19 vaccine in First Nations peoples are impacted by comorbidities
Source: Nat Immunol. 2023 May 29;24(6):966–78. doi: 10.1038/s41590-023-01508-y (PMC10232372; doi:10.1038/s41590-023-01508-y)
Supplement: Supplementary file 1 — Supplementary Tables 1–6. [file 41590_2023_1508_MOESM1_ESM.pdf]

nature immunology

Article

https://doi.org/10.1038/s41590-023-01508-y

Robust and prototypical immune responses toward COVID-19 vaccine in First Nations peoples are impacted by comorbidities

In the format provided by the authors and unedited

Supplementary Table 1. Demographic and clinical information of COVAC participants

	Study ID	Age	Gender	Ethnicity	COVID vaccine	Comorbidities
Australian First Nations	C013	48	female	Torres Strait Islander	BNT162b2	No
	C018	21	male	Aboriginal	BNT162b2	No
	C020	62	female	Aboriginal	BNT162b2	No
	C022	23	female	Aboriginal	BNT162b2	No
	C023	31	male	Aboriginal	BNT162b2	No
	C025	44	female	South Sea Islander	BNT162b2	No
	C026	50	male	Aboriginal	BNT162b2	Yes
	C028	50	female	Aboriginal	BNT162b2	No
	C033	44	female	Aboriginal	BNT162b2	No
	C035	58	female	Aboriginal	BNT162b2	Yes
	C036	71	female	Aboriginal	BNT162b2	Yes
	C037	50	male	Aboriginal	BNT162b2	Yes
	C038	79	female	Aboriginal	BNT162b2	Yes
	C042	63	female	Aboriginal	BNT162b2	Yes
	C044	51	female	Aboriginal	BNT162b2	Yes
	C046	47	male	Aboriginal & Torres Strait Islander	BNT162b2	No
	C049	56	female	Aboriginal	BNT162b2	Yes
	C050	50	female	Aboriginal	BNT162b2	Yes
	C063	46	male	Aboriginal	BNT162b2	Yes
	C064	47	male	Aboriginal	BNT162b2	Yes
	C065	52	female	Aboriginal	BNT162b2	Yes
	C071	58	male	Aboriginal	BNT162b2	Yes
	C072	57	male	Aboriginal	BNT162b2	Yes
	C073	22	male	Aboriginal	BNT162b2	No
	C074	54	female	Aboriginal	BNT162b2	Yes
	C075	24	male	Aboriginal	BNT162b2	No
	C076	31	male	Aboriginal & Torres Strait Islander	BNT162b2	No
	C077	62	male	Aboriginal	BNT162b2	No
	C078	60	male	Aboriginal	BNT162b2	No
	C079	34	male	Aboriginal	BNT162b2	No
	C080	21	female	Aboriginal	BNT162b2	No
	C081	55	male	Aboriginal	BNT162b2	Yes
	C082	67	male	Aboriginal	BNT162b2	Yes
	C084	48	female	Aboriginal	BNT162b2	No
	C087	21	male	Aboriginal	BNT162b2	No
	C088	19	female	Aboriginal	BNT162b2	No
	C089	58	male	Aboriginal	BNT162b2	No
	C090	37	female	Aboriginal	BNT162b2	No
	C091	36	male	Aboriginal	BNT162b2	No
	C092	34	male	Aboriginal	BNT162b2	No
	C093	31	male	Aboriginal	BNT162b2	No
	C094	42	female	Aboriginal	BNT162b2	No
	C095	31	female	Aboriginal	BNT162b2	No
	C096	34	female	Aboriginal	BNT162b2	No
	C097	49	male	Aboriginal	BNT162b2	Yes
	C098	30	female	Aboriginal	BNT162b2	No
	C099	37	male	Aboriginal	BNT162b2	Yes
	C100	52	female	Aboriginal	BNT162b2	No
	C101	19	male	Aboriginal	BNT162b2	Yes
	C102	39	male	Aboriginal	BNT162b2	No
	C103	43	female	Aboriginal	BNT162b2	No
	C104	60	female	Aboriginal	BNT162b2	Yes
	C105	32	male	Aboriginal	BNT162b2	Unknown
	C106	28	female	Aboriginal	BNT162b2	No
	C107	20	male	Aboriginal	BNT162b2	No
	C108	24	male	Aboriginal	BNT162b2	No
	C109	25	male	Aboriginal	BNT162b2	No
	C110	21	male	Aboriginal	BNT162b2	No
non-Indigenous	C001	45	female	Caucasian	BNT162b2	No
	C002	62	female	Caucasian	BNT162b2	No
	C003	43	female	Caucasian	BNT162b2	No
	C004	48	female	Caucasian	BNT162b2	No
	C005	42	female	Caucasian	BNT162b2	No
	C006	59	female	Caucasian	BNT162b2	No
	C007	43	female	Caucasian	BNT162b2	No
	C008	59	male	Caucasian	BNT162b2	No
	C009	44	female	Caucasian	BNT162b2	No
	C010	43	female	Caucasian	BNT162b2	No
	C011	59	female	Caucasian	BNT162b2	No
	C012	27	female	Caucasian	BNT162b2	No

C014	45	female	Caucasian	BNT162b2	No
C015	30	female	Caucasian	BNT162b2	No
C016	56	female	Caucasian	BNT162b2	No
C017	57	female	Caucasian	BNT162b2	No
C019	36	female	Caucasian	BNT162b2	No
C021	43	male	Caucasian	BNT162b2	No
C024	41	female	Caucasian	BNT162b2	No
C027	40	female	Caucasian	BNT162b2	No
C029	48	female	Caucasian	BNT162b2	No
C030	48	male	Caucasian	BNT162b2	No
C031	39	female	Caucasian	BNT162b2	No
C032	30	male	Caucasian	BNT162b2	No
C034	64	male	Caucasian	BNT162b2	No
C041	41	female	Asian	BNT162b2	No
C043	46	female	Caucasian	BNT162b2	No
C045	48	male	Caucasian	BNT162b2	No
C048	23	male	Caucasian	BNT162b2	No
C051	60	female	Caucasian	BNT162b2	No
C052	28	female	Caucasian	BNT162b2	No
C053	45	male	Caucasian	BNT162b2	No
C054	39	male	Caucasian	BNT162b2	No
C055	49	male	Asian	BNT162b2	No
C056	59	female	Caucasian	BNT162b2	No
C057	30	female	Caucasian	BNT162b2	No
C060	29	female	Asian	BNT162b2	No
C067	49	female	Caucasian	BNT162b2	No
C068	29	female	Caucasian	BNT162b2	No

Supplementary Table 2. Demographic and clinical information of additional non-Indigenous cohorts with comorbidities

Study ID	Age	Gender	Ethnicity	Renal disease	Diabetes	Inflammatory Bowel Disease	COVID-19 vaccination
D01	44	male	Caucasian	No	Yes	NA	No
D02	38	female	Caucasian	No	Yes	NA	No
D03	43	male	Caucasian	Yes	No	NA	No
D04	44	female	Caucasian	Yes	Yes	NA	No
D05	55	male	Caucasian	Yes	Yes	NA	No
D06	46	female	Non-Indigenous	Yes	No	NA	No
D07	59	male	Caucasian	Yes	Yes	NA	No
D08	61	male	Caucasian	No	Yes	NA	No
D09	68	male	Caucasian	Yes	No	NA	No
D10	65	female	Caucasian	No	Yes	NA	No
D11	53	male	Caucasian	Yes	No	NA	No
D12	44	male	Caucasian	Yes	No	NA	No
D13	73	female	Caucasian	Yes	Yes	NA	No
D14	68	female	Caucasian	No	Yes	NA	No
D15	39	female	Non-Indigenous	Yes	No	NA	No
D16	72	female	Caucasian	No	Yes	NA	No
D17	63	male	Caucasian	Yes	No	NA	ChAdOx1-S
D18	47	female	Caucasian	Yes	No	NA	BNT162b2
D19	58	male	Caucasian	Yes	No	NA	BNT162b2
D20	45	male	Caucasian	Yes	No	NA	BNT162b2
D21	58	female	Caucasian	Yes	No	NA	BNT162b2
D22	53	male	Caucasian	Yes	Yes	NA	BNT162b2
D23	67	male	Caucasian	Yes	Yes	NA	BNT162b2
D24	65	male	Non-Indigenous	No	Yes	NA	ChAdOx1-S
D25	61	male	Indian	No	Yes	NA	ChAdOx1-S
D26	70	female	Non-Indigenous	No	Yes	NA	ChAdOx1-S
D27	76	female	Non-Indigenous	No	pre-Diabetes	NA	BNT162b2
D28	57	male	African	No	Yes	NA	BNT162b2
D29	73	male	Caucasian	No	Yes	NA	BNT162b2
D30	45	male	Caucasian	No	Yes	NA	BNT162b2
D31	69	female	South Asian	No	Yes	NA	ChAdOx1-S
D32	57	male	Caucasian	Yes	Yes	NA	ChAdOx1-S
D33	62	male	Caucasian	Yes	No	NA	ChAdOx1-S
D34	62	male	Caucasian	Yes	No	NA	ChAdOx1-S
D35	27	female	Caucasian	No	Yes	NA	BNT162b2
D36	57	male	Caucasian	No	Yes	NA	ChAdOx1-S
D37	58	female	Caucasian	No	Yes	NA	BNT162b2
D38	62	female	Caucasian	No	Yes	NA	BNT162b2

I01	40	female	Non-Indigenous	NA	NA	Yes	BNT162b2
I02	49	female	Non-Indigenous	NA	NA	Yes	BNT162b2
I03	21	male	Non-Indigenous	NA	NA	Yes	BNT162b2
I04	21	male	Non-Indigenous	NA	NA	Yes	BNT162b2
I05	43	male	Non-Indigenous	NA	NA	Yes	BNT162b2
I06	30	male	Non-Indigenous	NA	NA	Yes	BNT162b2
I07	27	male	Non-Indigenous	NA	NA	Yes	BNT162b2
I08	30	male	Non-Indigenous	NA	NA	Yes	BNT162b2
I09	32	male	Non-Indigenous	NA	NA	Yes	BNT162b2
I10	24	male	Non-Indigenous	NA	NA	Yes	BNT162b2
I11	28	female	Non-Indigenous	NA	NA	Yes	BNT162b2
I12	26	male	Non-Indigenous	NA	NA	Yes	BNT162b2
I13	29	female	Non-Indigenous	NA	NA	Yes	BNT162b2
I14	53	female	Non-Indigenous	NA	NA	Yes	BNT162b2
I15	32	female	Non-Indigenous	NA	NA	Yes	BNT162b2
I16	50	female	Non-Indigenous	NA	NA	Yes	BNT162b2
I17	25	male	Non-Indigenous	NA	NA	Yes	BNT162b2
I18	54	male	Non-Indigenous	NA	NA	Yes	BNT162b2
I19	25	male	Non-Indigenous	NA	NA	Yes	BNT162b2
I20	30	male	Non-Indigenous	NA	NA	Yes	BNT162b2
I21	54	female	Non-Indigenous	NA	NA	Yes	BNT162b2
I22	16	male	Non-Indigenous	NA	NA	Yes	BNT162b2
I23	34	female	Non-Indigenous	NA	NA	Yes	BNT162b2
I24	33	female	Non-Indigenous	NA	NA	Yes	BNT162b2
I25	47	female	Non-Indigenous	NA	NA	Yes	BNT162b2
I26	22	female	Non-Indigenous	NA	NA	Yes	BNT162b2
I27	27	female	Non-Indigenous	NA	NA	Yes	BNT162b2
I28	36	female	Non-Indigenous	NA	NA	Yes	BNT162b2
I29	28	male	Non-Indigenous	NA	NA	Yes	BNT162b2
I30	20	female	Non-Indigenous	NA	NA	Yes	BNT162b2
I31	64	female	Non-Indigenous	NA	NA	Yes	ChAdOx1-S

Supplementary Table 3. HLA profile of Australian First Nations participants in the COVAC cohort

Study ID	HLA-A	HLA-B	HLA-C	HLA-DRB1	HLA-DPB1	HLA-DQB1
C013	24:02:01, 32:01:01	44:02:01, 55:02:01	05:01:01, 08:01:01	04:04:01, 09:01:02	04:01:01, 05:01:01	03:02:01G, 03:03:02
C018	02:01:01, 24:02:01	07:02:01, 56:02:01	01:02:01, 07:02:01	04:05:01, 15:01:01	04:01:01, 05:01:01	04:02:01, 06:02:01
C020	01:01, 34:01	07:02, 15:21	04:03, 07:02	14:08, 15:01	04:01, 05:01	05:03, 06:02
C023	01:01:01, 68:02:01	14:02:01, 18:01:01	07:01:01, 08:02:01	11:04:01, 13:03:01	02:01:02, 04:01:01	03:01:01G
C025	03:01:01, 24:02:01	18:01:01, 57:01:01	06:02:01, 07:01:01	07:01:01, 15:02:01G	02:01:02, 13:01:01	03:03:02, 06:01:01G
C028	01:01:01, 02:01:01	08:01:01	07:01:01	03:01:01	01:01:01, 09:01:01	02:01:01G
C033	34:01:01	40:02:01, 56:01:01	01:02:01, 07:02:01	08:03:02	02:01:02, 04:01:01	06:01:01G
C037	34:01:01	13:01:01, 40:01:02	03:03:01, 04:01:01	08:03:02	02:01:02, 04:01:01	06:01:01G
C038	01:01:01, 34:01:01	08:01:01, 13:01:01	04:01:01, 07:01:01	03:01:01, 08:03:02	04:01:01, 09:01:01	02:01:01G, 06:01:01G
C042	03:01:01, 34:01:01	07:02:01, 40:02:01	12:03:01, 15:02:01	07:01:01, 12:01:01G	04:01:01, 05:01:01	03:01:01G, 03:03:02
C044	11:01:01, 24:02:01	13:01:01, 15:06	04:01:94, 04:03:01	04:05:01, 08:03:02	04:01:01	03:01:01G, 06:01:01G
C046	02:01, 11:01	07:02, 15:21	04:03, 07:02	04:05, 15:01	04:01	03:01, 06:02
C049	33:03:01, 34:01:01	15:21:01, 58:01:01	03:02:02, 04:03:01	14:04:01, 14:09	04:01:01, 22:01:01	04:02:01, 05:03:01
C050	02:01:01, 34:01:01	56:01:01, 56:02:01	01:02:01, 07:02:01	08:03:02	02:01:02, 05:01:01	06:01:01G
C063	34:01:01	15:25:01, 56:01:01	01:02:01	04:05:01, 08:03:02	04:01:01, 05:01:01	04:01:01, 06:01:01G
C065	11:01:01, 34:01:01	13:01:01	04:01:01	04:05:01, 08:03:02	02:01:02, 04:01:01	03:01:01G, 06:01:01G
C081	11:01:01, 34:01:01	40:01:02, 56:02:01	01:02:01, 03:03:01	08:03:02	02:01:02, 05:01:01	06:01:01G
C082	34:01:01	40:02:01	15:02:01	08:03:02, 12:01:01G	02:01:02	04:02:01, 06:01:01G
C084	34:01:01	40:02:01	01:02:01, 15:02:01	14:08, 14:10	02:01:02, 652:01	04:02:01
C085	11:01:01, 24:02:01	13:01:01, 44:02:01	04:01:94, 16:04:01	04:05:01, 11:04:01	04:01:01	03:01:01G
C087	11:01:01, 24:02:01	13:01:01, 56:01:01	04:01:01, 07:02:01	12:01:01G, 14:08	02:01:02, 04:01:01	04:02:01, 05:03:01
C088	02:01:01, 34:01:01	13:01:01, 56:01:01	04:01:01, 07:02:01	08:03:02	02:01:02, 04:01:01	06:01:01G
C089	11:01:01	13:01:01	04:01:01, 04:01:94	04:05:01, 14:10	02:01:02, 05:01:01	03:01:01G, 04:02:01
C090	11:01:01, 34:01:01	13:01:01	04:01:01	08:03:02, 14:54:01	02:01:02, 04:01:01	05:03:01, 06:01:NEW

C091	34:01:01	13:01:01	04:01:01	08:03:02	04:01:01	06:01:01G
C092	11:01:01, 34:01:01	13:01:01, 56:01:01	04:01:01, 07:02:01	08:03:02, 12:01:01G	02:01:02, 04:01:01	04:02:01, 06:01:01G
C093	02:01:01, 34:01:01	40:01:02, 56:01:01	03:03:01, 07:02:01	08:03:02	02:01:02	05:03:01, 06:01:01G
C094	11:01:01, 34:01:01	13:01:01	04:01:01	08:03:02, 14:08	04:01:01, 22:01:01	05:03:01, 06:01:01G
C096	11:01:01, 34:01:01	13:01:01, 56:60	04:01:01	15:02:01G	04:01:01	06:01:01G
C097	02:01:01, 24:02:01	13:01:01, 56:01:01	04:01:01, 07:02:01	04:05:01, 08:03:02	02:01:02	03:01:01G, 06:01:01G
C098	11:01:01	13:01:01	04:01:01, 04:01:94	04:05:01, 14:54:01	04:01:01	03:01:01G, 05:03:01
C099	11:01:01, 34:01:01	13:01:01, 56:60	04:01:01	08:03:02, 15:02:01G	02:01:02, 04:01:01	06:01:01G
C100	11:01:01	13:01:01, 56:01:01	04:01:01	04:12, 08:03:02	02:01:02, 05:01:01	04:02:01, 06:01:01G

Supplementary Table 4. Demographic, clinical and sampling information of Australian First Nations COVID-19 patients

			Days post disease onset										
Study ID	Age	Gender	V1	V2	Hospital admission	Hospital discharge	Comorbidities	COVID Vaccine brand	COVID Vaccine status	Treatments	Days in hospital	ICU requirement	Oxygen supply
L02	33	M	2		0	33	No	BNT162b2	2 doses	Budesonide Inhaler	33	Ward	None
L06	48	M	3	12	0	16	Yes	BNT162b2	2 doses	Azithromycin, Baricitinib, Dexamethasone, Meropenum, Remdesivir, Vancomycin	16	ICU	Non-invasive
L09	68	M	1	8	1	13	Yes	BNT162b2	3 doses	Dexamethasone, Remdesivir	12	Ward	Non-invasive
L10	55	M	6	11	4	11	Yes	BNT162b2	2 doses	Budesonide Inhaler, Dexamethasone, Salbutamol	7	Ward	Non-invasive
L11	69	F	8	11	0	17	Yes	BNT162b2	2 doses	Augmentin, Ceftriaxone, Doxycycline, Prednisolone	17	Ward	None
L12	66	F	4	9	0	11	Yes	BNT162b2	3 doses	Dexamethasone, Remdesivir	11	Ward	Non-invasive

Supplementary Table 5. TCR clonotypes of tetramer-specific T cells from Australian First Nations COVID-19 vaccines and patients

DP4/S_{167}

TRAV	TRAJ	CDR3a	TRBV	TRBJ	TRBD	CDR3b	C0 13 V3	C0 13 V4	C0 18 V1	C0 18 V3	C0 23 V3	C0 23 V4	C0 37 V3	C0 44 V3	C0 46 V1	C0 46 V3	C0 49 V3	C0 49 V4	L 02 V1	L 06 V1	L 06 V2	L 09 V2	L 10 V1	L 10 V2	L 11 V1	L 11 V2	L 12 V1	L 12 V2
TRAV35	TRAJ42	CASMNYGGSQGNLIF	TRBV6-2/TRBV6-3	TRBJ1-5	TRBD1	CASSSRGGQPQHF	1																					
TRAV35	TRAJ42	CAAMNYGGSQGNLIF	TRBV10-3	TRBJ1-2	TRBD2	CATAPGAGGLRGYTF		1																				
TRAV35	TRAJ42	CAGQLFGGSQGNLIF	ND	ND	ND	ND		1																				
TRAV38-1	TRAJ57	CAFMKPFITQGGSEKLVF	TRBV19	TRBJ2-3	TRBD1	CASRSTGAGTQYF			1																			
TRAV6	TRAJ9	CALNTGGFKTIF	TRBV2	TRBJ2-1	TRBD2	CASSRLAGGSNEQFF			1																			
TRAV35	TRAJ42	CAGQNYGGSQGNLIF	TRBV18	TRBJ1-2	TRBD1	CASSPRRAMTLLAGGYGYTF				3																		
TRAV9-2	TRAJ17	CALSESNKAAGNKLTF	TRBV9	TRBJ1-2	TRBD1	CASSARDRVAGYTF				2																		
ND	ND	ND	TRBV10-2	TRBJ1-2	TRBD1	CASSDRGALGYTF				1																		
ND	ND	ND	TRBV30	TRBJ1-4	TRBD1	CAWRGGDRVEKLFF				1																		
ND	ND	ND	TRBV6-1	TRBJ1-2	TRBD2	CASRRDSFGYTF				1																		
ND	ND	ND	TRBV6-2/TRBV6-3	TRBJ1-2	TRBD1	CASSYSRGSPSGYTF				1																		
ND	ND	ND	TRBV9	TRBJ1-2	TRBD1	CASSARDRVAGYTF				1																		
TRAV16	TRAJ52	CALWSAGGTSYGKLTF	TRBV4-1	TRBJ2-5	TRBD2	CASSQDPREATQYF				1																		
TRAV35	TRAJ17	CAGQLYKAAGNKLTF	TRBV6-1	TRBJ1-5	TRBD2	CASSGRESQPQHF				1																		
TRAV35	TRAJ42	CAGFNYGGSQGNLIF	TRBV3-1/TRBV3-2	TRBJ1-2	NA	CASSHSLGYTF				1																		
TRAV35	TRAJ42	CAGLNYGGSQGNLIF	TRBV10-3	TRBJ1-2	TRBD1	CATTERTGKVGGYTF				1																		
TRAV35	TRAJ42	CAGQLYGGSQGNLIF	ND	ND	ND	ND				1																		
TRAV35	TRAJ42	CAGQLYGGSQGNLIF	TRBV14	TRBJ1-2	TRBD2	CASTSRGGFGYTF				1																		
TRAV35	TRAJ42	CAGQNYGGSQGNLIF	TRBV15	TRBJ1-4	TRBD1	CATSRGTGTGGYWSNEKLFF				1																		
TRAV35	TRAJ42	CAGSNYGGSQGNLIF	TRBV9	TRBJ2-3	TRBD2	CASSPSGGSPDTQYF				1																		
TRAV35	TRAJ42	CAGVNYGGSQGNLIF	ND	ND	ND	ND				1																		
TRAV35	TRAJ42	CAPMNYGGSQGNLIF	TRBV11-2	TRBJ1-2	TRBD1	CASSARTGNVYGYTF				1																		
TRAV35	TRAJ42	CAGQNYGGSQGNLIF	ND	ND	ND	ND					3	1							1							4	2	
ND	ND	ND	TRBV6-6	TRBJ2-7	TRBD2	CASKKREGDFEQYF					2																	
ND	ND	ND	TRBV7-2	TRBJ1-5	TRBD1	CASSPGQGLQPQHF					2																	
TRAV35	TRAJ17	CAGQLFKAAGNKLTF	TRBV5-4/TRBV5-8	TRBJ2-5	TRBD1	CASSLRQGGETQYF					1	1																
TRAV35	TRAJ42	CAARNYGGSQGNLIF	TRBV24-1	TRBJ2-3	TRBD2	CATSGHYGLAGDTQYF					1	1																
TRAV35	TRAJ42	CAGLNYGGSQGNLIF	ND	ND	ND	ND					1								1				1			1		2
TRAV35	TRAJ17	CAGQLFKAAGNKLTF	TRBV6-2/TRBV6-3	TRBJ1-2	TRBD1	CASSRTGGNGYTF					1															1		
ND	ND	ND	TRBV18	TRBJ1-2	TRBD1	CASSPSTDPLGYTF					1																	
ND	ND	ND	TRBV20-1	TRBJ2-3	TRBD2	CSASSSGRLRAYF					1																	
ND	ND	ND	TRBV29-1	TRBJ1-2	TRBD1	CSVVSGIGYTF					1																	
ND	ND	ND	TRBV5-5	TRBJ1-2	TRBD2	CASSWSLGYTF					1																	
ND	ND	ND	TRBV6-1	TRBJ2-3	TRBD1	CASSARTGSDTQYF					1																	
ND	ND	ND	TRBV6-2/TRBV6-3	TRBJ1-1	TRBD2	CASSARGTEAFF					1																	
ND	ND	ND	TRBV7-2	TRBJ1-4	TRBD1	CASSLRTTNEKLFF					1																	
ND	ND	ND	TRBV7-2	TRBJ2-4	TRBD2	CASSQTGASGNHF					1																	
ND	ND	ND	TRBV7-2	TRBJ2-5	TRBD1	CASSSRQGSGETQYF					1																	
ND	ND	ND	TRBV7-8	TRBJ1-2	TRBD1	CASSVSQGTLYGYTF					1																	
ND	ND	ND	TRBV7-8	TRBJ2-1	TRBD1	CASSNGQGAFEQFF					1																	
ND	ND	ND	TRBV9	TRBJ2-2	TRBD1	CASSSRQAVNTGELFF					1																	
TRAV16	TRAJ37	CAPKHGSSNTGKLIF	TRBV7-2	TRBJ1-2	TRBD1	CASSPGPGALNGYTF					1																	
TRAV35	TRAJ17	CAGLLYKAAGNKLTF	TRBV6-6	TRBJ2-2	TRBD2	CASLRREGTGELFF					1																	
TRAV35	TRAJ17	CAGQLAKAAGNKLTF	TRBV27	TRBJ1-2	TRBD1	CASSLRGLNGYTF					1																	
TRAV35	TRAJ17	CAGQLFKAAGNKLTF	TRBV29-1	TRBJ2-5	TRBD1	CSAIHRGPETQYF					1																	
TRAV35	TRAJ17	CAGQLMKAAGNKLTF	ND	ND	ND	ND					1																	
TRAV35	TRAJ17	CAGQLMKAAGNKLTF	TRBV18	TRBJ2-5	TRBD2	CASSPRDSGETQYF					1																	
TRAV35	TRAJ17	CAGQLYKAAGNKLTF	TRBV9	TRBJ1-3	TRBD2	CASSPREGSGNTIYF					1																	
TRAV35	TRAJ42	CAALNYGGSQGNLIF	TRBV24-1	TRBJ1-2	TRBD1	CATSGTGGDIRGYTF					1																	
TRAV35	TRAJ42	CAGANYGGSQGNLIF	TRBV11-2	TRBJ2-5	TRBD1	CASSGRQGPPETQYF					1																	
TRAV35	TRAJ42	CAGFNYGGSQGNLIF	TRBV25-1	TRBJ2-1	TRBD1	CASRRTGGLNEQFF					1																	
TRAV35	TRAJ42	CAGLNYGGSQGNFIF	ND	ND	ND	ND					1																	
TRAV35	TRAJ42	CAGLNYGGSQGNLIF	TRBV19	TRBJ1-2	TRBD1	CASTGKGGALYGYTF					1																	
TRAV35	TRAJ42	CAGLNYGGSQGNLIF	TRBV5-4/TRBV5-8	TRBJ1-2	TRBD1	CASTTRGSLYGYTF					1																	
TRAV35	TRAJ42	CAGLNYGGSQGNLIF	TRBV6-1	TRBJ1-2	TRBD1	CASSAAGKLYGYTF					1																	
TRAV35	TRAJ42	CAGLNYGGSQGNLIF	TRBV6-1	TRBJ1-2	TRBD1	CASSAPGTLYGYTF					1																	
TRAV35	TRAJ42	CAGLNYGGSQGNLIF	TRBV6-2/TRBV6-3	TRBJ1-2	TRBD1	CASSYPGQLYGYTF					1																	
TRAV35	TRAJ42	CAGMNYGGSQGNLIF	TRBV20-1	TRBJ2-7	TRBD1	CSARKRWGINEQYF					1																	
TRAV35	TRAJ42	CAGMNYGGSQGNLIF	TRBV28	TRBJ1-2	TRBD1	CASSDRQGGGLSGYTF					1																	
TRAV35	TRAJ42	CAGQNYGGSQGNLIF	TRBV16	TRBJ1-2	TRBD1	CASSQSRGPIYGYTF					1																	
TRAV35	TRAJ42	CAGQNYGGSQGNLIF	TRBV6-1	TRBJ1-2	TRBD1	CASIKNKGALNGYTF					1																	
TRAV35	TRAJ42	CAGQNYGGSQGNLIF	TRBV9	TRBJ2-2	TRBD1	CASSLRHKTNTGELFF					1																	
TRAV35	TRAJ42	CAGQNYGGSQGNLIF	TRBV9	TRBJ2-2	TRBD1	CASSPKTPVNTGELFF					1																	
TRAV35	TRAJ42	CAGSNYGGSQGNLIF	TRBV7-8	TRBJ2-1	TRBD2	CASSLRRDPLNEQFF					1																	
TRAV35	TRAJ53	CAGQNSGGSNYKLTF	TRBV5-4	TRBJ2-2	TRBD1	CASSYSTGPLNTGELFF					1																	
TRAV6	TRAJ53	SGKGSNYKLTF	TRBV9	TRBJ2-7	TRBD2	CASSARTGLAGSSYEQYF					1																	
TRAV9-2	TRAJ35	CALRPIGFGNVLHC	TRBV30	TRBJ1-2	TRBD1	CAWSPPDRINYGYTF					1																	
TRAV9-2	TRAJ50	CALLKTSYDKVIF	TRBV20-1	TRBJ1-5	TRBD1	CSATTPGQGNQPQHF					1																	
ND	ND	ND	TRBV11-2	TRBJ2-3	TRBD2	CASSSRTSGPPETQYF						1																
ND	ND	ND	TRBV12-3/TRBV12-4	TRBJ1-3	TRBD1	CASSRSRDRFFSGNTIYF						1																
ND	ND	ND	TRBV7-2	TRBJ1-2	TRBD1	CASSKEPGQGGYGYTF						1																
TRAV35	TRAJ17	CAGFLFKAAGNKLTF	TRBV14	TRBJ1-2	TRBD1	CASSHRGDGYTF						1																
TRAV35	TRAJ17	CAGQLMKAAGNKLTF	TRBV5-6	TRBJ1-5	TRBD1	CASSSVGTEAENSNQPQHF						1																
TRAV35	TRAJ17	CAGQLSRAAGNKLTF	ND	ND	ND	ND						1																
TRAV35	TRAJ42	CAGLNYGGSQGNLIF	TRBV14	TRBJ1-2	TRBD2	CASSPRGGHGYTF						1																
TRAV35	TRAJ42	CAGLNYGGSQGNLIF	TRBV14	TRBJ1-2	TRBD2	CASSQRTGGLGYGYTF						1																
TRAV35	TRAJ42	CAGLNYGGSQGNLIF	TRBV9	TRBJ1-2	TRBD1	CASTRGMQGALSSNYGYTF						1																
TRAV35	TRAJ42	CAGMNYGGSQGNLIF	TRBV14	TRBJ1-2	TRBD2	CASSPRYGNLYGYTF						1																
TRAV35	TRAJ42	CAGQNYGGSQGNLIF	TRBV6-6	TRBJ1-2	TRBD1	CASSYRGDYGYTF						1																
TRAV35	TRAJ42	CAGVNYGGSQGNLIF	TRBV6-4	TRBJ2-2	TRBD2	CASSPREAGATGELFF						1																
TRAV35	TRAJ53	CAGQLSGGSNYKLTF	TRBV15	TRBJ2-2	TRBD1	CATSRALGPLNTGELFF						1																

TRAV35	TRAJ53	CAGQLSGGSNYKLTF	TRBV7-2	TRBJ1-2	TRBD1	CASSLTGNYGYTF	1													1		
ND	ND	ND	TRBV20-1	TRBJ2-3	TRBD2	CSAPKRDTQYF	1															
ND	ND	ND	TRBV9	TRBJ1-2	TRBD1	CASSVGTGGALNGYTF	1															
TRAV35	TRAJ17	CAGQLNKAAGNKLTF	TRBV11-2	TRBJ1-2	TRBD1	CASSLTGAYGYTF		1														
TRAV6	TRAJ53	CALEGSGGSNYKLTF	TRBV18	TRBJ1-1	TRBD1	CASSSGTGGYYTEAFF			1													
ND	ND	ND	TRBV11-2	TRBJ2-5	TRBD1	CASSPRQRGPPETQYF				1												
ND	ND	ND	TRBV6-5	TRBJ2-2	TRBD1	CASSKQHRPNTGELFF				1												
ND	ND	ND	TRBV6-5	TRBJ2-2	TRBD1	CASSYSTGRGPNTGELFF				1												
TRAV35	TRAJ42	CAGLNYGGSQGNLIF	TRBV6-6	TRBJ1-2	TRBD1	CASSYSAGGLGGYTF				1												
TRAV35	TRAJ42	CAGMNYGGSQGNLIF	TRBV9	TRBJ1-2	TRBD1	CASSRTGGKGYTF				1												
TRAV35	TRAJ42	CAGQNYGGSQGNLIF	TRBV14	TRBJ2-7	TRBD2	CASSPRTSGTGEQYF					2	2										
TRAV35	TRAJ42	CAGLNYGGSQGNLIF	TRBV5-5	TRBJ2-2	TRBD2	CASSLRLAGAGELFF					1	1										
TRAV35	TRAJ42	CAGMNYGGSQGNLIF	ND	ND	ND	ND					1			1			1		2	1	1	1
ND	ND	ND	TRBV19	TRBJ2-2	TRBD2	CASSGRAPTRTGELFF					1											
ND	ND	ND	TRBV2	TRBJ1-2	TRBD1	CASSDRGDGYTF					1											
ND	ND	ND	TRBV6-5	TRBJ1-1	TRBD1	CASINRGRTEAFF					1											
TRAV13-2	TRAJ52	CAEMSYAGGTSYGKLTF	TRBV28	TRBJ1-2	TRBD2	CASSARRGFGYTF					1											
TRAV22	TRAJ58	CAVYGVGETSGSRLTF	TRBV2	TRBJ2-1	TRBD2	CASRRWTSGSGMNEQFF					1											
TRAV35	TRAJ17	CAGQLHKAAGNKLTF	TRBV28	TRBJ1-3	TRBD1	CASTRRGNTIYF					1											
TRAV35	TRAJ32	CAGQNYGGATNKLIF	TRBV19	TRBJ2-2	NA	CASSIRFPANTGELFF					1											
TRAV35	TRAJ42	CAGLNYGGSQGNLIF	TRBV11-2	TRBJ1-3	TRBD1	CASSYRGSGNTIYF					1											
TRAV35	TRAJ42	CAGLNYGGSQGNLIF	TRBV14	TRBJ2-1	TRBD2	CASSQRTSGTGEQFF					1											
TRAV35	TRAJ42	CAGLNYGGSQGNLIF	TRBV9	TRBJ2-2	TRBD2	CASSPRLAGATGELFF					1											
TRAV35	TRAJ42	CAGQNYGGSQGNLIF	TRBV27	TRBJ2-2	TRBD2	CASSLRGRPNTGELFF					1											
TRAV35	TRAJ42	CAGQNYGGSQGNLIF	TRBV6-4	TRBJ1-2	TRBD1	CASTPRTGSPGGYTF					1											
TRAV35	TRAJ42	CAVMNYGGSQGNLIF	TRBV28	TRBJ1-2	TRBD2	CATSPRRGAVYGYTF					1											
ND	ND	ND	TRBV28	TRBJ1-2	TRBD1	CASSYRRGNGYTF						1										
TRAV35	TRAJ42	CAALNYGGSQGNLIF	ND	ND	ND	ND						1										
TRAV41	TRAJ49	CAVNHRNTGNQFYF	TRBV10-3	TRBJ2-5	TRBD1	CAIRARAPETQYF						1										
ND	ND	ND	TRBV6-1	TRBJ1-2	NA	CASSVSLGYTF							1									
ND	ND	ND	TRBV6-6	TRBJ1-2	TRBD1	CASSPRGQLYGYTF							1									
TRAV35	TRAJ42	CAAMNYGGSQGNLIF	TRBV6-1	TRBJ1-2	TRBD1	CASGVPGSPLDGYTF							1									
TRAV35	TRAJ42	CAGMNYGGSQGNLIF	TRBV10-3	TRBJ1-2	TRBD1	CAIRPGQGLGGYTF							1									
TRAV35	TRAJ42	CAGMNYGGSQGNLIF	TRBV6-1	TRBJ1-2	TRBD1	CASSGRQGTIYGYTF							1									
TRAV35	TRAJ42	CAGQNYGGSQGNLIF	TRBV6-5	TRBJ2-2	TRBD2	CASSYGRAGTGELFF							1									
TRAV35	TRAJ42	CAGRNYGGSQGNLIF	TRBV19	TRBJ1-2	TRBD1	CASSGSFGYTF							1									
TRAV35	TRAJ42	CAGVNYGGSQGNLIF	TRBV5-5	TRBJ2-2	TRBD2	CASSLRLAGATGELFF							1									
TRAV35	TRAJ42	CAYMNYGGSQGNLIF	ND	ND	ND	ND							1									
ND	ND	ND	TRBV6-2/TRBV6-3	TRBJ2-2	TRBD2	CASSYRGSGTGELFF								1								
TRAV26-1	TRAJ23	CIVRVRGNQGGKLIF	ND	ND	ND	ND								1								
TRAV35	TRAJ42	CAGFNYGGSQGNLIF	TRBV5-5	TRBJ1-2	TRBD2	CASSLRGADGYTF								1								
TRAV35	TRAJ42	CAGMNYGGSQGNLIF	TRBV6-2/TRBV6-3	TRBJ1-2	TRBD1	CASSSRGDYYGYTF								1								
TRAV35	TRAJ42	CAGQNYGGSQGNLIF	TRBV19	TRBJ1-2	TRBD2	CASTTRSGNLYGYTF								1								
TRAV35	TRAJ42	CAGQNYGGSQGNLIF	TRBV6-6	TRBJ1-2	TRBD1	CASSPRAGGLSGYTF								1								
TRAV35	TRAJ42	CASMNYGGSQGNLIF	TRBV19	TRBJ2-3	TRBD1	CASSMAPRGGFWATDTQYF								1								
ND	ND	ND	TRBV3-1/TRBV3-2	TRBJ2-5	TRBD1	CASRPSDTRGQTQYF									1							
ND	ND	ND	TRBV6-2/TRBV6-3	TRBJ1-2	TRBD1	CASSNRGFGYTF									1							
TRAV35	TRAJ17	CAGQLFRAAGNKLTF	TRBV3-1/TRBV3-2	TRBJ2-5	TRBD1	CASRPSDTRGQTQYF									1							
TRAV35	TRAJ42	CAALNYGGSQGNLIF	TRBV10-3	TRBJ1-2	TRBD1	CASRQGSLGYTF									1							
TRAV35	TRAJ42	CAALNYGGSQGNLIF	TRBV9	TRBJ2-2	TRBD1	CASSSRDRVNTGELFF									1							
TRAV35	TRAJ42	CAGHNYGGSQGNLIF	TRBV6-1	TRBJ1-2	TRBD1	CASSARQGSLSGYTF									1							
TRAV35	TRAJ42	CAGLNYGGSQGNLIF	TRBV6-1	TRBJ1-2	TRBD1	CASSQGQGSLGYTF									1							
TRAV35	TRAJ42	CAGLNYGGSQGNLIF	TRBV6-4	TRBJ1-4	TRBD1	CASSDRTGESATNEKLFF									1							
TRAV35	TRAJ42	CAGLNYGGSQGNLIF	TRBV9	TRBJ2-2	TRBD2	CASSPRLAGSTGELFF									1							
TRAV35	TRAJ42	CAGQNYGGSQGNLIF	TRBV19	TRBJ2-2	TRBD1	CASSIRNPANTGELFF									1							
TRAV35	TRAJ42	CAVMNYGGSQGNLIF	TRBV6-2/TRBV6-3	TRBJ1-2	TRBD1	CASSYRGTGLNYGYTF									1							
TRAV35	TRAJ42	CGGMNYGGSQGNLIF	ND	ND	ND	ND									1							
TRAV35	TRAJ17	CAGQLFKAAGNKLTF	TRBV27	TRBJ1-6	TRBD2	CASRGRGGSPLHF										1						
TRAV35	TRAJ42	CAAQNYGGSQGNLIF	TRBV6-6	TRBJ1-5	TRBD1	CASTARVGSPSSPQHF										1						
TRAV35	TRAJ42	CAGLNYGGSQGNLIF	TRBV5-5	TRBJ2-2	TRBD2	CASSPRGAGKTGELFF										1						
TRAV35	TRAJ17	CAGQLFKAAGNKLTF	TRBV18	TRBJ1-1	TRBD1	CASSPRGGEAFF											2					
TRAV35	TRAJ42	CATMNYGGSQGNLIF	ND	ND	ND	ND											2					
TRAV41	TRAJ34	CAVTPRNTDKLIF	TRBV6-2/TRBV6-3	TRBJ2-1	TRBD2	CASSRGGGLAGALSYNEQFF											1	1				
ND	ND	ND	TRBV10-1	TRBJ2-3	TRBD2	CASSGRGDTQYF											1					
ND	ND	ND	TRBV15	TRBJ1-2	TRBD1	CATSRDKNSLYGYTF											1					
ND	ND	ND	TRBV5-8	TRBJ1-2	TRBD1	CASSLRTGGLGYTF											1					
TRAV35	TRAJ42	CAALLYGGSQGNLIF	ND	ND	ND	ND											1					
TRAV35	TRAJ42	CAARNYGGSQGNLIF	TRBV10-2	TRBJ2-5	TRBD2	CASTPRAGVETQYF											1					
TRAV35	TRAJ42	CAGHLSGGSQGNLIF	TRBV7-2	TRBJ2-3	TRBD2	CASSPRRRDLSTDTQYF											1					
TRAV35	TRAJ42	CAGLNYGGSQGNLIF	TRBV14	TRBJ1-2	TRBD1	CASSSRDGALNGYTF											1					
TRAV35	TRAJ42	CAGLNYGGSQGNLIF	TRBV6-1	TRBJ1-2	TRBD1	CASSGKGGGLGGYTF											1					
TRAV35	TRAJ42	CAGLNYGGSQGNLIF	TRBV6-2/TRBV6-3	TRBJ1-2	TRBD1	CASSPRQGGFLYGYTF											1					
TRAV35	TRAJ42	CAGMNYGGSQGNLIF	TRBV6-1	TRBJ1-2	TRBD2	CASSRKGGAGMDGYTF											1					
TRAV35	TRAJ42	CAGQNYGGSQGNLIF	TRBV27	TRBJ2-1	TRBD1	CASSFKQGAFEQFF											1					
ND	ND	ND	TRBV7-2	TRBJ2-5	TRBD1	CASSSRRTGLETQYF												1				
TRAV35	TRAJ42	CAALNYGGSQGNLIF	TRBV6-6	TRBJ1-2	TRBD2	CASSPKGGLRGYTF												1				
ND	ND	ND	TRBV9	TRBJ2-2	TRBD1	CASSARQGVTGELFF													6	8		
TRAV35	TRAJ42	CAGQNYGGSQGNLIF	TRBV5-1	TRBJ2-2	TRBD2	CASSPRGVNTGELFF													2			
TRAV35	TRAJ42	CAGRNYGGSQGNLIF	TRBV11-2	TRBJ2-3	TRBD1	CASSLRGDTQYF													1	2		
ND	ND	ND	TRBV11-2	TRBJ2-3	TRBD1	CASSLRGDTQYF													1	1		
TRAV10	TRAJ43	CVVSARWHNDMRF	ND	ND	ND	ND													1			
TRAV35	TRAJ17	CAGQLFKAAGNKLTF	TRBV11-2	TRBJ2-5	TRBD2	CASGGRTSGETQYF													1			
TRAV35	TRAJ42	CAALNYGGSQGNLIF	TRBV5-6	TRBJ2-7	TRBD2	CASSSRRGGEQYF													1			
TRAV35	TRAJ42	CAGKNYGGSQGNLIF	TRBV11-2	TRBJ2-3	TRBD1	CASSLRGDTQYF													1			
TRAV35	TRAJ42	CAGLNYGGSQGNLIF	TRBV15	TRBJ1-2	TRBD1	CATSPRGRVYGYTF													1			
TRAV35	TRAJ42	CAGLNYGGSQGNLIF	TRBV6-2/TRBV6-3	TRBJ1-2	TRBD1	CASSNRGSFGYTF													1			
TRAV35	TRAJ42	CAGMNYGGSQGNLIF	TRBV9	TRBJ2-2	TRBD1	CASSVRQRPNTGELFF													1			
TRAV35	TRAJ42	CAGQNYGGSQGNLIF	TRBV6-2/TRBV6-3	TRBJ1-2	TRBD1	CASSYSQGALHGYTF													1			

TRAV35	TRAJ42	CAGQNYGGSQGNLIF	TRBV6-2/TRBV6-3	TRBJ1-2	TRBD1	CASSYSTGSVYGYTF																			1			
TRAV35	TRAJ42	CAGQNYGGSQGNLIF	TRBV9	TRBJ2-2	TRBD1	CASSVRDRANTGELFF																			1			
TRAV35	TRAJ42	CAVQNYGGSQGNLIF	ND	ND	ND	ND																			1			
TRAV35	TRAJ42	CAYMNYGGSQGNLIF	TRBV25-1	TRBJ1-2	TRBD2	CASSGAFGYTF																			1			
TRAV35	TRAJ53	CAGQLSGGSNYKLTF	ND	ND	ND	ND																			1			
TRAV36/DV7	TRAJ52	CAVVGNAGGTSYGKLTF	ND	ND	ND	ND																			1			
TRAV35	TRAJ17	CAGQLSKAAGNKLTF	TRBV27	TRBJ1-4	TRBD2	CASSLRGFLGKLFF																				2		
ND	ND	ND	TRBV27	TRBJ1-4	TRBD2	CASSLRGFLGKLFF																				1		
ND	ND	ND	TRBV30	TRBJ1-2	TRBD1	CASPRGVGYTF																				1		
TRAV10	TRAJ43	CVVSARWHNDMRF	TRBV7-2	TRBJ2-1	TRBD2	CASSSRTSGKYNEQFF																				1		
TRAV35	TRAJ17	CAGQLHKAAGNKLTF	TRBV18	TRBJ2-7	TRBD2	CASSPRGSEQYF																				1		
TRAV35	TRAJ17	CAGQLHRAAGNKLTF	TRBV7-2	TRBJ2-3	TRBD2	CASSLGSGSTQYF																				1		
TRAV35	TRAJ42	CAGKNYGGSQGNLIF	TRBV6-4	TRBJ1-1	TRBD1	CASSPRDGSLEAFF																				1		
TRAV35	TRAJ42	CAGLNYGGSQGNLIF	TRBV15	TRBJ1-2	TRBD1	CATSSRTGALVGYTF																				1		
TRAV35	TRAJ42	CAGLNYGGSQGNLIF	TRBV9	TRBJ2-2	TRBD1	CASSVRRLPNTGELFF																				1		
TRAV35	TRAJ42	CAGMNYGGSQGNLIF	TRBV28	TRBJ1-2	TRBD1	CASNRGGLGYTF																				1		
TRAV35	TRAJ42	CAGMNYGGSQGNLIF	TRBV6-2/TRBV6-3	TRBJ1-2	TRBD2	CASSYRGGDGYTF																				1		
TRAV35	TRAJ42	CAGQNYGGSQGNLIF	TRBV30	TRBJ1-2	TRBD2	CARLSGVGYTF																				1		
TRAV35	TRAJ42	CAGQNYGGSQGNLIF	TRBV6-5	TRBJ2-2	TRBD2	CASSYATSGRPNTGELFF																				1		
TRAV35	TRAJ42	CAGQNYGGSQGNLIF	TRBV6-5	TRBJ2-2	TRBD2	CASSYRAGLAGPNTGELFF																				1		
TRAV35	TRAJ42	CAGRNYGGSQGNLIF	ND	ND	ND	ND																				1		
TRAV35	TRAJ42	CAGVNYGGSQGNLIF	TRBV25-1	TRBJ1-2	TRBD1	CASSTGSPYGYTF																				1		
TRAV35	TRAJ42	CAGQNYGGSQGNLIF	TRBV6-1	TRBJ2-1	TRBD2	CASKGTSGSFEQFF																					2	1
TRAV35	TRAJ42	CAAQNYGGSQGNLIF	ND	ND	ND	ND																					1	1
TRAV35	TRAJ42	CAGMNYGGSQGNLIF	TRBV9	TRBJ2-3	TRBD1	CASSLRPGTDTQYF																					1	1
ND	ND	ND	TRBV5-5	TRBJ1-4	TRBD1	CASSLAYRDEKLFF																					1	
ND	ND	ND	TRBV6-1	TRBJ1-5	TRBD2	CAGGWREGQPQHF																					1	
ND	ND	ND	TRBV6-1	TRBJ2-3	TRBD1	SSQGSTDTQYF																					1	
ND	ND	ND	TRBV6-6	TRBJ2-1	TRBD1	CASSPRAGGFEQFF																					1	
ND	ND	ND	TRBV7-3	TRBJ1-2	TRBD2	CASSLNGNYGYTF																					1	
ND	ND	ND	TRBV9	TRBJ2-2	TRBD2	CASSPRGDPNTGELFF																					1	
TRAV26-2	TRAJ53	CILRDDSGGSNYKLTF	TRBV10-3	TRBJ1-2	TRBD1	CAISATGGSVNGYTF																					1	
TRAV35	TRAJ17	CAGQLHRAAGNKLTF	TRBV27	TRBJ2-1	TRBD1	CASSPRGDEQFF																					1	
TRAV35	TRAJ42	CAALNYGGSQGNLIF	TRBV25-1	TRBJ2-1	TRBD2	CASERRGSYNEQFF																					1	
TRAV35	TRAJ42	CAAMNYGGSQGNLIF	TRBV6-1	TRBJ1-2	TRBD1	CASSKQGSLSGYTF																					1	
TRAV35	TRAJ42	CAAMNYGGSQGNLIF	TRBV7-2	TRBJ1-2	TRBD1	CASSLGRGSIYGYTF																					1	
TRAV35	TRAJ42	CAAQNYGGSQGNLIF	TRBV11-2	TRBJ1-2	TRBD1	CASSFSQGSYGYTF																					1	
TRAV35	TRAJ42	CAGMNYGGSQGNLIF	TRBV11-2	TRBJ1-2	TRBD1	CASSTRQGAIDGYTF																					1	
TRAV35	TRAJ42	CAGMNYGGSQGNLIF	TRBV7-6	TRBJ2-1	TRBD1	CASSLVRGPAGRSSYNEQFF																					1	
TRAV35	TRAJ42	CAGQNYGGSQGNLIF	TRBV6-1	TRBJ1-2	TRBD1	CASSARQGPINGYTF																					1	
TRAV35	TRAJ42	CAGQNYGGSQGNLIF	TRBV6-2/TRBV6-3	TRBJ1-4	TRBD1	CASSYSGGTGKFF																					1	
TRAV35	TRAJ42	CAGQNYGGSQGNLIF	TRBV7-8	TRBJ1-3	TRBD1	CASSIRGTSGNTIYF																					1	
TRAV35	TRAJ42	CAGRNYGGSQGNLIF	TRBV14	TRBJ1-2	TRBD1	CASSQAWGSEYGYTF																					1	
TRAV35	TRAJ42	CAPQNYGGSQGNLIF	ND	ND	ND	ND																					1	
TRAV35	TRAJ42	CAVLNYGGSQGNLIF	ND	ND	ND	ND																					1	
ND	ND	ND	TRBV10-3	TRBJ1-2	TRBD1	CAISATGGSVNGYTF																						1
ND	ND	ND	TRBV6-1	TRBJ2-2	TRBD1	CASSSRQAATGELFF																						1
ND	ND	ND	TRBV9	TRBJ2-2	TRBD2	CASSPRGGPGTGELFF																						1
TRAV21	TRAJ52	IAGGTSYGKLTF	TRBV20-1	TRBJ2-3	TRBD2	CSARVYYSDTQYF																						1
TRAV35	TRAJ17	CAGQLARAAGNKLTF	TRBV19	TRBJ1-5	TRBD1	CASSRTGLLPQHF																						1
TRAV35	TRAJ17	CAGQLFKAAGNKLTF	TRBV6-2/TRBV6-3	TRBJ2-3	TRBD2	CASSSRADTQYF																						1
TRAV35	TRAJ17	CAGYLYKAAGNKLTF	TRBV6-1	TRBJ2-5	TRBD2	CASSERSGRVLETQYF																						1
TRAV35	TRAJ42	CAGHNYGGSQGNLIF	TRBV9	TRBJ2-2	TRBD1	CASSPRGQPNTGELFF																						1
TRAV35	TRAJ42	CAGLNYGGSQGNLIF	TRBV19	TRBJ1-2	TRBD1	CASSGRSGSLYGYTF																						1
TRAV35	TRAJ42	CAGLNYGGSQGNLIF	TRBV6-5	TRBJ1-2	TRBD1	CASSYSPGSLYGYTF																						1
TRAV35	TRAJ42	CAGMNYGGSQGNLIF	TRBV14	TRBJ1-2	TRBD1	CASSQGRGLGYTF																						1
TRAV35	TRAJ42	CAGMNYGGSQGNLIF	TRBV14	TRBJ1-2	TRBD2	CASSPRSGNGYTF																						1
TRAV35	TRAJ42	CAGQNYGGSQGNLIF	TRBV27	TRBJ1-2	TRBD1	CASSSRPGHPYGYTF																						1
TRAV35	TRAJ42	CAGQNYGGSQGNLIF	TRBV6-2/TRBV6-3	TRBJ1-5	TRBD1	CASSYSQGQPQHF																						1
TRAV35	TRAJ42	CAGRNYGGSQGNLIF	TRBV19	TRBJ1-2	TRBD2	CASSSRQGGFGYTF																						1
TRAV35	TRAJ53	CAGLNSGGSNYKLTF	TRBV18	TRBJ1-2	TRBD1	CASSPRTDWIYGYTF																						1
						Total	1	2	2	20	50	16	3	1	1	5	17	6	11	8	12	3	18	3	26	36	27	22

A2/S_{269}

TRAV	TRAJ	CDR3a	TRBV	TRBJ	TRBD	CDR3b	C018 V1	C018 V3	C028 V1	C028 V3	C028 V4	C028 V5	C046 V1	C046 V3	C046 V5	C050 V1	C097 V3	L06 V1	L06 V2
ND	ND	ND	TRBV29-1	TRBJ1-2	TRBD2	CSVEGESYGYTF	1												
ND	ND	ND	TRBV7-9	TRBJ2-7	NA	CASSDSYEQYF	1												
ND	ND	ND	TRBV13	TRBJ2-2	TRBD1	CASSEAQGGNTGELFF		1											
ND	ND	ND	TRBV20-1	TRBJ2-2	TRBD2	CSARDGRVWNTGELFF		1											
ND	ND	ND	TRBV7-9	TRBJ1-1	TRBD1	CASSPDIEAFF		1											
TRAV13-2	TRAJ34	CAEIGHTDKLIF	ND	ND	ND	ND		1											
TRAV4	TRAJ8	CLVDTGFQKLVF	TRBV24-1	TRBJ2-2	TRBD1	CATSDFAGDTGELFF		1											

TRAV9-2	TRAJ17	CALSVFQIKAAGNKLTF	TRBV20-1	TRBJ2-2	NA	CSARDLFSQNTGELFF	1				
TRAV12-3	TRAJ24	CAMSCDSWGKLQF	TRBV13	TRBJ2-5	TRBD1	CASSHGTGQETQYF		1			
TRAV35	TRAJ30	CAGVRDDKIIF	TRBV27	TRBJ2-7	TRBD2	GSSYEQYF		1			
ND	ND	ND	TRBV30	TRBJ2-2	TRBD1	CAWSPPALGNTGELFF			1		
TRAV9-2	TRAJ58	CALSIWGETSGSRLTF	TRBV12-3/TRBV12-4	TRBJ2-2	TRBD1	CATQSENTGELFF				3	1
ND	ND	ND	TRBV7-8	TRBJ2-2	TRBD2	CATGELDTGELFF				1	1
ND	ND	ND	TRBV12-3/TRBV12-4	TRBJ2-2	TRBD1	CAGQTANTGELFF				1	
ND	ND	ND	TRBV15	TRBJ2-2	TRBD1	CAVQILNTGELFF				1	
ND	ND	ND	TRBV19	TRBJ2-2	TRBD1	CATQDLNTGELFF				1	
TRAV17	TRAJ36	CATDPGTGANNLFF	TRBV7-9	TRBJ2-7	TRBD1	CASSPDIEQYF				1	
TRAV20	TRAJ32	CAVDYGGATNKLIF	TRBV13	TRBJ1-4	TRBD1	CASSLQTEKLFF				1	
TRAV38-1	TRAJ24	CAQHKTDSWGKLQF	TRBV5-1	TRBJ2-2	TRBD2	CASGDSNTGELFF				1	
TRAV5	TRAJ26	CAEDHGQNFVF	ND	ND	ND	ND				1	
TRAV27	TRAJ42	CAGASHMNYGGSQGNLIF	TRBV2	TRBJ2-2	NA	CASMVSLNTGELFF					3
ND	ND	ND	TRBV20-1	TRBJ2-2	TRBD1	CSARDPDAMNTGELFF					2
ND	ND	ND	TRBV7-9	TRBJ1-1	TRBD2	CASSPEIEAFF					2
ND	ND	ND	TRBV7-9	TRBJ2-2	TRBD1	CARSDPNTGELFF					2
TRAV13-1	TRAJ43	CAAEKYNNNDMRF	ND	ND	ND	ND					2
TRAV19	TRAJ36	CALSYTGANNLFF	TRBV20-1	TRBJ2-2	TRBD1	CSARDDRATNTGELFF					2
TRAV23/DV6	TRAJ48	ANFGNEKLTF	TRBV7-8	TRBJ2-7	TRBD1	CASSSGNEQYF					2
TRAV8-3	TRAJ33	CAVIMDSNYQLIW	TRBV11-1	TRBJ2-2	TRBD1	CAILFDRNTGELFF					2
ND	ND	ND	TRBV12-3/TRBV12-4	TRBJ2-2	TRBD2	CAAGSLNTGELFF					1
ND	ND	ND	TRBV2	TRBJ2-2	TRBD1	CAGNDQNTGELFF					1
ND	ND	ND	TRBV20-1	TRBJ2-2	TRBD1	CSARDDRATNTGELFF					1
ND	ND	ND	TRBV3-1/TRBV3-2	TRBJ2-2	TRBD1	CASQYANTGELFF					1
ND	ND	ND	TRBV7-8	TRBJ2-2	NA	CARENQNTGELFF					1
ND	ND	ND	TRBV7-9	TRBJ2-1	TRBD1	CASSPDIEQFF					1
ND	ND	ND	TRBV7-9	TRBJ2-7	TRBD1	CASSPDIEQYF					1
ND	ND	ND	TRBV7-9	TRBJ2-7	TRBD1	CASSPDISGYF					1
TRAV20	TRAJ15	CASGNQAGTALIF	TRBV28	TRBJ2-1	TRBD2	CATGLLNSNEQFF					1
TRAV27	TRAJ13	CADPNAGNNHIQNP	TRBV5-6	TRBJ2-2	TRBD1	CAVGDLNTGELFF					1
TRAV27	TRAJ38	CADPNAGNNRKLIW	TRBV5-6	TRBJ2-2	TRBD1	CAVGDLNTGELFF					1

TRAV36/DV7	TRAJ44	CARVHTGTASKLTF	TRBV7-8	TRBJ2-7	TRBD1	CASSLGYEQYF						1							
TRAV9-2	TRAJ29	CALTNSGNTPLVF	TRBV7-8	TRBJ2-2	TRBD1	CALQDGNTGELFF						1							
ND	ND	ND	TRBV6-2/TRBV6-3	TRBJ2-7	TRBD1	CASSYGDLPSGIEQYF							1						
TRAV1-1	TRAJ26	CAVESYGQNFVF	TRBV3-1/TRBV3-2	TRBJ2-5	TRBD2	CASSHAETSGKETQYF							1						
TRAV16	TRAJ12	CALMDSSYKLIF	TRBV29-1	TRBJ2-2	TRBD1	CSVDGPGFTNTGELFF							1						
ND	ND	ND	TRBV19	TRBJ2-2	TRBD2	CASSILAGGNTGELFF								3					
TRAV14/DV4	TRAJ49	CAMREKDTGNQFYF	TRBV9	TRBJ2-2	NA	CASSVPNTGELFF								3					
ND	ND	ND	TRBV19	TRBJ2-2	TRBD1	CAAQDLNTGELFF								2					
ND	ND	ND	TRBV20-1	TRBJ2-2	TRBD1	CSARDEPAVNTGELFF								2					
ND	ND	ND	TRBV2	TRBJ2-2	TRBD1	CASDDRNTGELFF								1	1				
ND	ND	ND	TRBV10-2	TRBJ2-2	TRBD1	CATHRDMNTGELFF								1					
ND	ND	ND	TRBV2	TRBJ2-2	NA	CASISLNNGELFF								1					
ND	ND	ND	TRBV2	TRBJ2-2	TRBD1	CAREGDRNTGELFF								1					
ND	ND	ND	TRBV2	TRBJ2-2	TRBD2	CAREGDRNTGELFF								1					
ND	ND	ND	TRBV20-1	TRBJ2-1	TRBD2	CSAPPGLEGDEQFF								1					
ND	ND	ND	TRBV20-1	TRBJ2-3	TRBD2	CSATDYRVTDTQYF								1					
ND	ND	ND	TRBV7-9	TRBJ2-7	TRBD1	CASSSDIVQYF								1					
TRAV12-1	TRAJ41	CVVNRVNSGYALNF	TRBV19	TRBJ2-2	TRBD2	CASSILAGGNTGELFF								1					
TRAV12-1	TRAJ43	CVVNELNDMRF	TRBV5-1	TRBJ2-1	TRBD2	CASSLDPEEQFF								1					
TRAV12-1	TRAJ43	CVVNRGNDMRF	TRBV2	TRBJ2-2	TRBD1	CASSELDSGELFF								1					
TRAV12-2	TRAJ43	CAVNRGNDMRF	TRBV19	TRBJ2-2	TRBD1	CAAQDLNTGELFF								1					
TRAV26-1	TRAJ20	CIASKLSF	TRBV6-1	TRBJ2-2	TRBD1	CATQVENTGELFF								1					
TRAV5	TRAJ21	CAEENNFNKFYF	TRBV20-1	TRBJ2-2	TRBD1	CSASGQVGRPGELFF										1			
ND	ND	ND	TRBV2	TRBJ2-2	NA	CASSDLNTGELFF											1		
TRAV23/DV6	TRAJ47	CAANPKGANMEYGNKLVF	TRBV13	TRBJ2-2	TRBD1	CASTPTGGGNTGELFF											1		
ND	ND	ND	TRBV15	TRBJ2-2	TRBD1	CATEHLDTGELFF												1	1
TRAV8-2/TRAV8-4	TRAJ38	CVVSLRGRMRGNNRKLIW	TRBV10-2	TRBJ2-2	TRBD2	CATLAEMNTGELFF												1	
ND	ND	ND	TRBV7-9	TRBJ2-7	NA	CASSLDIEQYF													1
						Total	2	6	2	1	11	32	3	23	1	1	2	2	2

A24/S_{1208}

TRAV	TRAJ	CDR3a	TRBV	TRBJ	TRBD	CDR3b	C013 V1	C013 V3	C018 V1	C018 V3	C025 V3	C097 V3	L10 V1	L11 V1	L11 V2	L12 V1	L12 V2
TRAV8-2/TRAV8-4	TRAJ33	CAVSGLPRVDSNYQLIW	TRBV24-1	TRBJ2-1	TRBD2	CATSQGTSPVDEQFF	1										

TRAV9-2	TRAJ42	CALARGGSQGNLIF	TRBV20-1	TRBJ2-7	TRBD1	CSARDLRTGGHYEQYF		1									
ND	ND	ND	TRBV11-2	TRBJ2-7	TRBD2	CASSFFTNEQYF			1								
ND	ND	ND	TRBV20-1	TRBJ1-1	TRBD1	CSARDLGNTEAFF			1								
TRAV16	TRAJ28	CALSVPGAGSYQLTF	TRBV28	TRBJ1-1	TRBD1	CASSLNVGYKAFF			1								
TRAV40	TRAJ53	CLLLTRGSGGSNYKLTF	TRBV12-5	TRBJ2-7	TRBD1	CASGLSASLGFPYEQYF			1								
ND	ND	ND	TRBV20-1	TRBJ2-7	TRBD2	CSARGLGAYEQYF				1							
ND	ND	ND	TRBV7-9	TRBJ2-1	TRBD1	CASSALGQQTSYNEQFF				1							
TRAV17	TRAJ32	CATDRRGGATNKLIF	TRBV24-1	TRBJ2-1	TRBD1	CATSVGQGATYEQFF				1							
TRAV38-1	TRAJ7	CACLIYGNNRLAF	TRBV7-2	TRBJ1-5	TRBD1	CASSLDRSNQPQHF				1							
TRAV4	TRAJ26	CLVGPFRDNYGQNFVF	TRBV13	TRBJ2-5	TRBD1	CASSPPDRGEQETQYF				1							
ND	ND	ND	TRBV15	TRBJ2-1	TRBD1	CATSRDQGNEQFF					1						
ND	ND	ND	TRBV20-1	TRBJ2-3	TRBD2	CSARDSNTDTQYF						1					
ND	ND	ND	TRBV20-1	TRBJ2-7	TRBD1	CSARSVGGGGGHYEQYF						1					
TRAV13-2	TRAJ58	EETSGSRLTF	TRBV20-1	TRBJ2-7	TRBD1	CSARSVGGGGGHYEQYF						1					
TRAV21	TRAJ22	CAVFSSGSARQLTF	TRBV20-1	TRBJ2-4	TRBD1	CSATKDRWDIQYF						1					
TRAV29/DV5	TRAJ28	CAASPYSGAGSYQLTF	TRBV5-6	TRBJ1-2	TRBD1	CASSLGRGQRGYTF						1					
TRAV4	TRAJ37	CLVGDPHGSSNTGKLIF	TRBV27	TRBJ2-1	TRBD2	CASSSRLAGQSYNEQFF						1					
ND	ND	ND	TRBV20-1	TRBJ2-7	TRBD1	CSARLNSAASVEQYF							1				
ND	ND	ND	TRBV4-1	TRBJ2-7	TRBD2	CASSQDSGGYYEQYF							1				
ND	ND	ND	TRBV7-8	TRBJ2-3	TRBD1	CASSLVQGVTDTQYF							1				
ND	ND	ND	TRBV10-1	TRBJ2-1	TRBD1	CASSEEGGQSDEQFF								1			
ND	ND	ND	TRBV20-1	TRBJ2-1	TRBD2	CSARDRGLAGVEQFF								1			
TRAV16	TRAJ26	CALDNYGQNFVF	TRBV5-6	TRBJ2-7	TRBD1	CASRPQGLYEQYF								1			
ND	ND	ND	TRBV20-1	TRBJ1-4	TRBD1	CSAVTIEGQRNEKLFF									1		
ND	ND	ND	TRBV27	TRBJ2-3	TRBD2	CASSLVAGGTDTQYF									1		
TRAV29/DV5	TRAJ26	CAARRDNYGQNFVF	ND	ND	ND	ND										3	1
TRAV16	TRAJ33	CALSGIGDSNYQLIW	TRBV5-6	TRBJ2-7	TRBD1	CASSLAGSFYEQYF										1	
TRAV4	TRAJ42	CLAVDGGSQGNLIF	TRBV20-1	TRBJ2-7	TRBD2	CSARVLLTSGRLGINEQYF										1	
TRAV41	TRAJ34	CAVRKYNTDKLIF	TRBV6-1	TRBJ2-7	TRBD1	CASSERGRGVLYEQYF										1	
ND	ND	ND	TRBV6-2/TRBV6-3	TRBJ2-4	TRBD1	CASSYSQREWGGNIQYF											1
TRAV24	TRAJ53	CAPRSGGSNYKLTF	ND	ND	ND	ND											1
						Total	1	1	4	5	1	6	3	3	2	6	3

Supplementary Table 6. Primers of Multiplex-nested RT-PCR

TRAV External	TRAV External Sequence	TRBV External	TRBV External Sequence	TRAV Internal	TRAV Internal Sequence	TRBV Internal	TRBV Internal Sequence
huTRAV1ext	AACTGCACGTACCAGACATC	huTRBV2ext	TCGATGATCAATTCTCAGTTG	huTRAV1int	GCACCCACATTTCTKTCTTAC	huTRBV2int	TTCACTCTGAAGATCCGGTC
huTRAV2ext	GATGTGCACCAAGACTCC	huTRBV3ext	CAAAATACCTGGTCACACAG	huTRAV2int	CACTCTGTGTCCAATGCTTAC	huTRBV3int	AATCTTCACATCAATTCCCTG
huTRAV3ext	AAGATCAGGTCAACGTTGC	huTRBV4ext	TCGCTTCTCACCTGAATG	huTRAV3int	ATGCACCTATTCAGTCTCTGG	huTRBV4int	CCTGCAGCCAGAAGACTC
huTRAV4ext	CTCCATGGACTCATATGAAGG	huTRBV5-1_4ext	GATTCTCAGGKCKCCAGTTC	huTRAV4int	ATTATATCACGTGGTACCAACAG	huTRBV5-1_4int	CTTGGAGCTGGRSGACTC
huTRAV5ext	CTTTTCCTGAGTGTCCGAG	huTRBV5-5_8ext	GTACCAACAGGYCCTGGGT	huTRAV5int	TACACAGACAGCTCCTCCAC	huTRBV5-5_8int	TCTGAGCTGAATGTGAACG
huTRAV6ext	CACCCTGACCTGCAACTATAC	huTRBV6-1_3,5_9ext	ACTCAGACCCCAAAATTCC	huTRAV6int	TGGTACCGACAAGATCCAG	huTRBV6-1_3,5_9int	GTGTRCCCAGGATATGAACC
huTRAV7ext	GCAAAATACAGGGATGGG	huTRBV6-4ext	ACTGGCAAAGGAGAAGTCC	huTRAV7int	TATGAGAAGCAGAAAGGAAGAC	huTRBV6-4int	TGGTTATAGTGTCTCCAGAGC
huTRAV8-1ext	CTCACTGGAGTTGGGATG	huTRBV7-1_3ext	TRTGATCCAATTTCAGGTCA	huTRAV8-1int	GTCAACACCTTCAGCTTCTC	huTRBV7-1_3int	TCYACTCTGAMGWTCCAGCG
huTRAV8-3ext	CACTGTCTCTGAAGGAGCC	huTRBV7-4_9ext	CGSWTCTYTGCAGARAGGC	huTRAV8-3int	TTTGAGGCTGAATTTAAGAGG	huTRBV7-4_9int	TGRMGATYCAGCGCACA
huTRAV8-2,4ext	GCCACCCTGGTTAAAGG	huTRBV9ext	GATCACAGCAACTGGACAG	huTRAV8-2,4int	AGAGTGAAACCTCCTTCCAC	huTRBV9int	GTACCAACAGAGCCTGGAC
huTRAV8-6ext	GAGCTGAGGTGCAACTACTC	huTRBV10-1ext	CAGAGCCCAAGACACAAG	huTRAV8-6int	AACCAAGGACTCCAGCTTC	huTRBV10-1int	TGGTATCGACAAGACCTGG
huTRAV8-7ext	CTAACAGAGGCCACCCAG	huTRBV10-2ext	ACCTTGATGTGTCACCAGAC	huTRAV8-7int	ATCAGAGGTTTTGAGGCTG	huTRBV10-3int	GGAACACCAGTGACTCTGAG
huTRAV9-1_2ext	TGGTATGTCCAATATCCTGG	huTRBV11ext	CGATTTTCTGCAGAGACGC	huTRAV9-1_2int	GAAACCACTTCTTTCCACTTG	huTRBV11int	GACTCCACTCTCAAGATCCA
huTRAV10ext	CAAGTGGAGCAGAGTCCTC	huTRBV12ext	ARGTGACAGARATGGGACAA	huTRAV10int	GAAAGAACTGCACTCTTCAATG	huTRBV12int	CYACTCTGARGATCCAGCC
huTRAV12-1_3ext	CARTGTTCCAGAGGGAGC	huTRBV13ext	AGCGATAAAGGAAGCATCC	huTRAV12-1_3int	AAGATGGAAGGTTTACAGCAC	huTRBV13int	CATTCTGAACTGAACATGAGC
huTRAV13-1ext	CATCCTTCAACCCTGAGTG	huTRBV14ext	CCAACAATCGATTCTTAGCTG	huTRAV13-1int	TCAGACAGTGCCTCAAACTAC	huTRBV14int	ATTCTACTCTGAAGGTGCAGC
huTRAV13-2ext	CAGCGCCTCAGACTACTTC	huTRBV15ext	AGTGACCCTGAGTTGTTCTC	huTRAV13-2int	CAGTGAAACATCTCTCTCTGC	huTRBV15int	ATAACTTCCAATCCAGGAGG
huTRAV14ext	AAGATAACTCAAACCCAACCAG	huTRBV16ext	GTCTTTGATGAAACAGGTATGC	huTRAV14int	AGGCTGTGACTCTGGACTG	huTRBV16int1	CTGTAGCCTTGAGATCCAGG
huTRAV16ext	AGTGGAGCTGAAGTGCAAC	huTRBV17ext	CAGACCCCCAGACACAAG	huTRAV16int	GTCCAGTACTCCAGACAACG	huTRBV17int	TGTTCACTGGTACCGACAG
huTRAV17ext	GGAGAAGAGGATCCTCAGG	huTRBV18ext	CATAGATGAGTCAGGAATGCC	huTRAV17int	CCACCATGAACTGCAGTTAC	huTRBV18int	CGATTTTCTGCTGAATTTCC
huTRAV18ext	TCCAGTATCTAAACAAAGAGCC	huTRBV19ext	AGTTGTGAACAGAATTTGAACC	huTRAV18int	TGACAGTTCCTTCCACCTG	huTRBV19int	TTCCTCTCACTGTGACATCG
huTRAV19ext	AGGTAACTCAAGCGCAGAC	huTRBV20ext	AAGTTTCTCATCAACCATGC	huTRAV19int	TGTGACCTTGGACTGTGTG	huTRBV20int	ACTCTGACAGTGACCAGTGC

huTRAV20ext	CACAGTCAGCGGTTTAAGAG	huTRBV23ext	GCGATTCTCATCTCAATGC	huTRAV20int	TCTGGTATAGGCAAGATCCTG	huTRBV23int	GCAATCCTGTCCTCAGAAC
huTRAV21ext	TTCCTGCAGCTCTGAGTG	huTRBV24ext	CCTACGGTTGATCTATTACTCC	huTRAV21int	AACTTGGTTCTCAACTGCAG	huTRBV24int	GATGGATACAGTGTCTCTCGA
huTRAV22ext	GTCCTCCAGACCTGATTCTC	huTRBV25ext	ACTACACCTCATCCACTATTCC	huTRAV22int	CTGACTCTGTGAACAATTTGC	huTRBV25int	CAGAGAAGGGAGATCTTTCC
huTRAV23ext	TGCTTATGAGAACACTGCG	huTRBV27,28ext	TGGTATCGACAAGACCCAG	huTRAV23int	TGCATTATTGATAGCCATACG	huTRBV27,28int	TTCYCCCTGATYCTGGAGTC
huTRAV24ext	CTCAGTCACTGCATGTTCAG	huTRBV29ext	TTCTGGTACCGTCAGCAAC	huTRAV24int	TGCCTTACACTGGTACAGATG	huTRBV29int	TCTGACTGTGAGCAACATGAG
huTRAV25ext	GGACTTCACCACGTACTGC	huTRBV30ext	TCCAGCTGCTCTTCTACTCC	huTRAV25int	TATAAGCAAAGGCCTGGTG	huTRBV30int	AGAATCTCTCAGCCTCCAGAC
huTRAV26-1ext	GCAAACCTGCCTTGTAATC	huTRBCext	TAGAACTGGACTTGACAGCG	huTRAV26-1int	CGACAGATTCACTCCCAG	huTRBCint	TTCTGATGGCTCAAACACAG
huTRAV26-2ext	AGCCAAATTCAATGGAGAG			huTRAV26-2int	TTCACTTGCCTTGTAACCAC		
huTRAV27ext	TCAGTTTCTAAGCATCCAAGAG			huTRAV27int	CTCACTGTGTACTGCAACTCC		
huTRAV29ext	GCAAGTTAAGCAAAATTCACC			huTRAV29int	CTGCTGAAGGTCCTACATTC		
huTRAV30ext	CAACAACCAGTGCAGAGTC			huTRAV30int	AGAAGCATGGTGAAGCAC		
huTRAV34ext	AGAACTGGAGCAGAGTCCTC			huTRAV34int	ATCTCACCATAAACTGCACG		
huTRAV35ext	GGTCAACAGCTGAATCAGAG			huTRAV35int	ACCTGGCTATGGTACAAGC		
huTRAV36ext	GAAGACAAGGTGGTACAAAGC			huTRAV36int	ATCTCTGGTTGTCCACGAG		
huTRAV38ext	GCACATATGACACCAGTGAG			huTRAV38int	CAGCAGGCAGATGATTCTC		
huTRAV39ext	CTGTTCCTGAGCATGCAG			huTRAV39int	TCAACCACTTCAGACAGACTG		
huTRAV40ext	GCATCTGTGACTATGAACTGC			huTRAV40int	GGAGGCGGAAATATTAAAGAC		
huTRAV41ext	AATGAAGTGGAGCAGAGTCC			huTRAV41int	TTGTTTATGCTGAGCTCAGG		
huTRACext	GACCAGCTTGACATCACAG			huTRACint	TGTTGCTCTTGAAGTCCATAG		
