# Supplementary material for: Robust and prototypical immune responses toward COVID-19 vaccine in First Nations peoples are impacted by comorbidities
Source: Nat Immunol. 2023 May 29;24(6):966–78. doi: 10.1038/s41590-023-01508-y (PMC10232372; doi:10.1038/s41590-023-01508-y)
Supplement: Supplementary file 2 — Reporting Summary [file 41590_2023_1508_MOESM2_ESM.pdf]

nature portfolio

Corresponding author(s): Katherine Kedzierska, Jane Davies, Thi H O Nguyen

Last updated by author(s): Mar 31, 2023

Reporting Summary

Nature Portfolio wishes to improve the reproducibility of the work that we publish. This form provides structure for consistency and transparency in reporting. For further information on Nature Portfolio policies, see our Editorial Policies and the Editorial Policy Checklist.

Statistics

For all statistical analyses, confirm that the following items are present in the figure legend, table legend, main text, or Methods section.

n/a	Confirmed	
☐	☒	The exact sample size (*n*) for each experimental group/condition, given as a discrete number and unit of measurement
☐	☒	A statement on whether measurements were taken from distinct samples or whether the same sample was measured repeatedly
☐	☒	The statistical test(s) used AND whether they are one- or two-sided<br>*Only common tests should be described solely by name; describe more complex techniques in the Methods section.*
☐	☒	A description of all covariates tested
☐	☒	A description of any assumptions or corrections, such as tests of normality and adjustment for multiple comparisons
☐	☒	A full description of the statistical parameters including central tendency (e.g. means) or other basic estimates (e.g. regression coefficient) AND variation (e.g. standard deviation) or associated estimates of uncertainty (e.g. confidence intervals)
☐	☒	For null hypothesis testing, the test statistic (e.g. *F*, *t*, *r*) with confidence intervals, effect sizes, degrees of freedom and *P* value noted<br>*Give P values as exact values whenever suitable.*
☒	☐	For Bayesian analysis, information on the choice of priors and Markov chain Monte Carlo settings
☒	☐	For hierarchical and complex designs, identification of the appropriate level for tests and full reporting of outcomes
☐	☒	Estimates of effect sizes (e.g. Cohen's *d*, Pearson's *r*), indicating how they were calculated

Our web collection on statistics for biologists contains articles on many of the points above.

Software and code

Policy information about availability of computer code

Data collection	BD FACS DIVA v8.0.1; Thermo Ascent Software for Multiskan v2.4; LabChip GXII Touch HT Microchip-CE platform
Data analysis	FlowJo v10; Prism v9; R v4.2.0, v4.2.1, ggalluvial v0.12.3, Inkscape v1.2, psych v2.2.5, rstatix v0.7.0, corrplot v0.92, EnhancedVolcano v1.14.0; online QOGNIT LEGENDplex™ program; LabChip GX Touch software v1.9.1010.0; LabChip GX Reviewer software v5.4.2222.0, TCRdist v0.0.2, TCRdist3 v0.2.2, R package NLME v3.1-160, bowtie2 program v2.5.0, mixcr v3.0.13

For manuscripts utilizing custom algorithms or software that are central to the research but not yet described in published literature, software must be made available to editors and reviewers. We strongly encourage code deposition in a community repository (e.g. GitHub). See the Nature Portfolio guidelines for submitting code & software for further information.

Data

Policy information about availability of data

All manuscripts must include a data availability statement. This statement should provide the following information, where applicable:

- Accession codes, unique identifiers, or web links for publicly available datasets
- A description of any restrictions on data availability
- For clinical datasets or third party data, please ensure that the statement adheres to our policy

The published article includes all datasets generated or analyzed during the study. Source data are provided with this paper as Source Date files. TCR sequences in this study were uploaded to Mendeley Data, with the access code DOI: 10.17632/fj636xh5y6.1. Raw FACS data are shown in the manuscript. FACS-source files are available from the authors upon request. The majority data of the current study are based on flow cytometry and there are hundreds of FACS-source files with a big file size. We are happy to provide any single file upon request.

Field-specific reporting

Please select the one below that is the best fit for your research. If you are not sure, read the appropriate sections before making your selection.

☒ Life sciences ☐ Behavioural & social sciences ☐ Ecological, evolutionary & environmental sciences

For a reference copy of the document with all sections, see nature.com/documents/nr-reporting-summary-flat.pdf

Life sciences study design

All studies must disclose on these points even when the disclosure is negative.

Sample size	The sample size was determined by the availability of samples from Australian First Nations and non-Indigenous participants that received the BNT162b2 COVID-19 vaccine in Northern Territory, Australia in 2021-2022. The sample size of non-Indigenous participants with comorbidities (renal disease, diabetes, IBD) was determined by the availability of samples, with recruitment performed through larger cohorts.
Data exclusions	No data were excluded with the following exception which was pre-established: donors who had a total number of less than 10 counted tetramer+CD8+ or tetramer+CD4+ T cells within the whole enriched fraction were excluded for further phenotypic analyses as cell numbers were too low (Fig 5h). This was indicated in the manuscript in Methods. 4 samples were excluded from spike-specific B cell analyses due to minimal numbers of lymphocytes or CD19+ B cells. A source data file was generated to show all data points.
Replication	Experiments could not be replicated due to limited PBMC numbers. These are rare and unique patient samples and so we were limited to performing all the available assays. To ensure reliability, all timepoints from the same patient were carried out in the same experiment.
Randomization	Randomization was not applicable to the study, participants received BNT162b2 or ChAdOx1-S COVID-19 vaccines following the current vaccine recommendations in Australia.
Blinding	Experiments were not blinded as specific experiments were designed for COVID-19 vaccinees, for example HLA type was needed to perform tetramer studies.

Reporting for specific materials, systems and methods

We require information from authors about some types of materials, experimental systems and methods used in many studies. Here, indicate whether each material, system or method listed is relevant to your study. If you are not sure if a list item applies to your research, read the appropriate section before selecting a response.

Materials & experimental systems

n/a	Involved in the study	
☐	☒	Antibodies
☐	☒	Eukaryotic cell lines
☒	☐	Palaeontology and archaeology
☒	☐	Animals and other organisms
☐	☒	Human research participants
☒	☐	Clinical data
☒	☐	Dual use research of concern

Methods

n/a	Involved in the study	
☒	☐	ChIP-seq
☐	☒	Flow cytometry
☒	☐	MRI-based neuroimaging

Antibodies

Antibodies used

We used commercially-available antibodies as per Material and Methods.
AIM assay: anti-CXCR5-BV421 (562747; BD Biosciences; clone RF8B2), anti-CD3-BV510 (317332; BioLegend; clone OKT3), anti-CD8-BV605 (564116; BD Biosciences; clone SK1), anti-CD4-BV650 (563875; BD Biosciences; clone SK3), anti-CD25-BV711 (563159; BD Biosciences; clone 2A3), anti-CXCR3-BV785 (353738; BioLegend; clone G025H7), anti-CD137-APC (309810; BioLegend; clone 4B4-1), anti-CD27-AF700 (560611; BD Biosciences; clone M-T271), anti-CD14/CD19-APC-H7 (560180 clone MϕP9/560252 clone SJ25C1; BD Biosciences), anti-CD69-PerCPCy5.5 (310925; BioLegend; clone FN50), anti-CD134-PE (340420; BD Biosciences; clone L106), anti-CD95-PE-CF594 (562395; BD Biosciences; clone DX2), anti-CD45RA-PeCy7 (337167; BD Biosciences; clone L48).
ICS assay: anti-CD3-BV510 (317332; BioLegend; clone OKT3), anti-CD4-BV650 (563875; BD Biosciences; clone SK3), anti-CD8-PerCPCy5.5 (565310; BD Biosciences; clone SK1) anti-IFNg-v450 (560371; BD Biosciences; clone B27), anti-MIP-1b-APC (560656; BD Biosciences; clone D21-1351) anti-TNF-AF700 (557996; BD Biosciences; clone MAb11).
Tetramer enrichment assay: anti-CD71-BV421 (#562995; BD Biosciences; clone M-A712), anti-CD4-BV650 (#563875; BD Biosciences; clone SK3), anti-CD27-BV711 (#563167; BD Biosciences; clone L128), anti-CD38-BV786 (#563964; BD Biosciences; clone HIT2), anti-CCR7-AF700 (#561143; BD Biosciences; clone 150503), anti-CD14-APC-H7 (#560180; BD Biosciences; clone MϕP9), anti-CD19-APC-H7 (#560177; BD Biosciences; clone SJ25C1), anti-CD45RA-FITC (#555488; BD Biosciences; clone HI100), anti-CD8-PerCP-Cy5.5 (#565310; BD Biosciences; clone SK1), anti-CD95-PE-CF594 (#562395; BD Biosciences; clone DX2), anti-PD1-PE-Cy7 (#561272; BD Biosciences; clone EH12.1), anti-CD3-BV510 (#317332; Biolegend; clone OKT3), anti-HLA-DR-BV605 (#307640; Biolegend; clone L243)

Validation

All antibodies were obtained from commercial vendors. Each antibody used had a validated technical data sheet as per manufacturer's website showing positive staining, and titrated in our laboratory to define the appropriate concentration prior to their use. ELISA assay were tested with samples with known high responses from previous assays. FACS positive staining is shown in the FACS plots in the main figures.

Eukaryotic cell lines

Policy information about cell lines

Cell line source(s)

Vero cells were obtained from ATCC (#CCL-81).

Authentication

The cell line was not authenticated.

Mycoplasma contamination

Vero cells tested mycoplasma negative.

Commonly misidentified lines (See ICLAC register)

No commonly misidentified cell lines were used in the study.

Human research participants

Policy information about studies involving human research participants

Population characteristics

Please refer to Supplementary Tables 1-4 for details.

Recruitment

Samples were recruited through the Menzies School of Health research in Darwin, Northern Territory; Alfred Hospital; Royal Melbourne Hospital; Austin Hospital; Adelaide Health Network; La Trobe University and University of Melbourne, Australia. Participants were recruited from a wide range of settings including but not limited to: healthcare and university staff vaccination programs, land council community events, remote community vaccination days, hospital and health services campus including a community based dialysis unit. Overall presence of co-morbidities was consistent with reported rates for First Nations people in the Northern Territory. Signed informed consents were obtained from all blood donors prior to the study.

Ethics oversight

Experiments conformed to the Declaration of Helsinki Principles and the Australian National Health and Medical Research Council Code of Practice. Written informed consent was obtained from all blood donors prior to the study. The study was approved by the the Human Research Ethics Committee of the Northern Territory Department of Health and Menzies School of Health Research (#2012-1928, COVAC, LIFT), The Alfred Hospital (#280-14, DISI), the Royal Melbourne Hospital (Melbourne, Australia) with approval from Melbourne Health (HREC/74403/MH-2021 and HREC/17/MH/53), Central Adelaide Local Health Network Human Research Ethics Committee; CALHN (HREC 14541; recruitment was part of the REVAX trial (ACTRN12621000532808), La Trobe Human Ethics Committee (HEC21097), Austin Hospital Ethics Committee (HREC/75984/Austin-2021) and the University of Melbourne Human Research Ethics Committees (#11077, #21864 and #11124, #15398-3).

Note that full information on the approval of the study protocol must also be provided in the manuscript.

Flow Cytometry

Plots

Confirm that:

☒ The axis labels state the marker and fluorochrome used (e.g. CD4-FITC).

☒ The axis scales are clearly visible. Include numbers along axes only for bottom left plot of group (a 'group' is an analysis of identical markers).

☒ All plots are contour plots with outliers or pseudocolor plots.

☒ A numerical value for number of cells or percentage (with statistics) is provided.

Methodology

Sample preparation

Samples were prepared as described in Methods. Peripheral blood was collected in heparinised or EDTA tubes and serum tubes, with plasma and sera collected after centrifugation, respectively. Peripheral blood monocular cells (PBMCs) were isolated via Ficoll-Paque separation.

Instrument

BD LSRII Fortessa, BD FACSAriaIII or BD FACSCanto II was used for acquisition of data

Software

BD FACS Diva v8.0.1, FlowJo v10

Cell population abundance

Only single cell sorting was performed, which was confirmed by the presence of single TCR chains.

Gating strategy

Gating strategy has been described in the figures, figure legends and Extended Data Figure 2.
The Activation-Induced Markers Assay starts with Lymphocyte gate based on FSC-A and SSC-A, then Singlet gate by FSC-A and FSC-H, then a Time gate to ensure signal consistency. Live T cells were gated on CD3+DUMP- cells (DUMP composed of Live

or Dead, CD14, CD19). CD4+ and CD8+ T cells were gated on CD4+CD8- or CD4-CD8+ cells respectively, followed by gating on CD134+CD137+ cells or CD69+CD137+ cells respectively. CXCR5+CD4+ T follicular helper (TFH), CXCR5+CXCR3+ TFH1, CXCR5 +CXCR3- TFH2 or 17, CXCR5-CD4+ T helper (TH), CXCR5-CXCR3+ TH1, CXCR5-CXCR3- TH2 or 17 cells were also gated for CD134+CD137+ expression.
The Intracellular Cytokine Staining Assay starts with Lymphocyte gate based on FSC-A and SSC-A, then Singlet gate by FSC-A and FSC-H, then a Time gate to ensure signal consistency. Live T cells were gated on Live or Dead-CD3+ cells. CD4+ and CD8+ T cells were gated on CD4+CD8- or CD4-CD8+ cells respectively, followed by gating on IFN-γ+TNF+ cells.
The Tetramer enrichment Assay starts with Lymphocyte gate based on FSC-A and SSC-A, then Singlet gate by FSC-A and FSC-H. Live T cells were gated on CD3+DUMP- cells (DUMP composed of Live or Dead, CD14, CD19). CD4+ and CD8+ T cells were gated on CD4+CD8- or CD4-CD8+ cells respectively. CD4+ and CD8+ T cells were then gated on tetramer staining, followed by further phenotyping including CD27+CD45RA+CCR7+CD95- Naive T cells, CD27+CD45RA+CD95+ TSCM cells, CD27+CD45RA- TCM-like cells, CD27-CD45RA- TEM-like cells, CD27-CD45RA+ TEMRA cells.

☒ Tick this box to confirm that a figure exemplifying the gating strategy is provided in the Supplementary Information.
